# Incurable

**Published:** 1872-02

**Authors:** 


### INCURABLE.

It must be a dreadful thing to be poor and sick, and with a sickness, too, which must remain, long or short, for life. Reader, bless the Infinite One from the very bottom of your heart, if this is not your condition; and to prove your sincerity, resolve this moment, and carry out the resolution on the spot, to do something for those who are old, and poor, and sick, and have no home; and for the unfortunate young, upon whom the sun of worldly prosperity has never shone, and who are, in addition, destined already to a tedious death from suffering, and pain, and anguish, from which there is no surcease till the saddened spirit returns to the God who gave it. There are men and women who have given their influence, their time, their minds, and their sympathizing tears, to secure a beautiful home for such, — the incurable poor. Let nothing hinder you from joining in a work so noble, so divine; and send a contribution which shall be a liberal one, for you, as a New Year's present to humanity.

It is not an obscure "Institution" by any means, nor is it in feeble hands; among its managers are some of our most distinguished merchants and bankers, men of the first business capacity, who give their special personal attention as a guarantee that every dollar, every penny, shall be expended to the greatest advantage, and in a way to do the most good. Send your money, in the shape of a post-office order or check, payable to J. D. Vermilye, Merchants' National Bank, New York, at 42 Wall Street. If you would like to see this establishment, and should happen to be in New York, starting from the front of the Astor House, take the Third Avenue cars to Tremont, one mile from the "Home;" or the Fourth Avenue cars, which take you to the Grand Union Depot in 42d Street, from which point the steam-cars will take you to West Farms, where the "Home" is located. During 1871 fifty-seven unfortunates received the protecting aid of the Institution. Those who are able are expected to pay, according to their ability, up to a reasonable remuneration; those who have nothing are in the name of God, whose children they are, made welcome.

SIXTEEN FREE BEDS

are there, and they are always occupied. A free bed entitles the occupant to a home there as long as he lives; no fear of ever being turned out to die. The beds are thus made free: some noble men or women give a certain amount of money to the Institution, to be invested securely, so that it can never be lost, the interest only to be expended for the support of the sufferer. If it is one, three, five thousand dollars, then there are sixty, a hundred and eighty, or three hundred dollars a year, which shall shelter, and feed, and nurse a suffering sister or brother man, for all time.

The bed may be called by the donor's name, which will be held in blessed remembrance by suffering occupants for ages after the graveyard monument has gone to decay, and the little earth mound reduced to a common level with the surrounding dust. What great heart shall open itself and purse-strings at the same time, and thus make for some poor sufferer

AN IMPERISHABLE BED.

To show with what confidence the management of the "Home" is looked upon by its near neighbors, the late William Summerfield, of Tremont, New York, left to it the sum of five thousand dollars.

It is worthy of notice that by far the greater number, in fact three times the number of any other ailment in the Institution, is

HEMIPLEGIA, that is, a paralysis of half the body, arising from some form of inflammation of the brain, or derangement of the nervous system. Nervous diseases are becoming alarmingly more frequent among all classes of persons, especially since the war. It takes so much more to make a fortune nowadays.

A man, to have a fortune, must possess at least three times as much as twenty years since; it requires three times the energy to make a living, and men in their hurry and struggles, their desperations to bring about their ends, so tax their bodies and their brains, as to overdraw on their vitality; and as a result, in thousands of cases, they are stricken down in a moment with a paralysis of some portion of the body, to be an affliction for life. The best means for avoiding any form of paralysis is to live temperately, regularly; obtain abundant sleep; "let your moderation be known unto all men;" curb excesses in living — the appetites and passions of our nature; in whatever business you may be engaged, pursue it calmly, steadily; repress all false, all worldly ambition, which impels you to efforts beyond your strength; in doing these things, you will find a blessing alway.

If you have no money to bestow on such a charity, send whatever is good to eat, or drink, or wear, or enjoy. To give you an idea, we take the "donations" in the order acknowledged in the last (fifth) annual report. One map, one can of peaches, one package of sea moss farine, one basket of pears, fifty fresh eggs, two cans each of peaches and tomatoes, three beefsteaks, half a barrel of potatoes, a basket of apples, and other things of money's worth, and just as good as the money; besides these, late papers, and magazines, and books, and pictorials, are thankfully received. No doubt the Secretary, Henry M. McLaren, 124 East 78th Street, will take charge of whatever may be sent for the benefit of "The Home of Incurables."

